# New Avenues for Prevention of Work-Related Diseases Linked to Psychosocial Risks

**DOI:** 10.3390/ijerph182111354

**Published:** 2021-10-28

**Authors:** Michel P. Guillemin

**Affiliations:** Faculty of Biology and Medicine, University of Lausanne, CH-1005 Lausanne, Switzerland; michel.guillemin@unil.ch

**Keywords:** occupational health, psychosocial risks, work, governance, work-related diseases

## Abstract

The epidemic of psychosocial risks continues to increase and the COVID-19 pandemic has even worsened this threat on workers’ health. This inexorable and evidence-based rise seems to be impervious to the preventive strategies proposed for more than 40 years. Hypotheses are proposed to explain this serious problem that drastically impacts public health and the economy. The objectives of this paper are to present, in this broad context of societal and cultural changes, how the present shift in management paradigms may represent opportunities to reduce work-related diseases. In the first part of this paper, we will summarize the situation on three main issues and their relation with psychosocial risks: (1) evolution of the occupational safety and health field, (2) change in the nature of work, and (3) emerging models of governance. In the second part, we will describe, through a few examples (among many others), how emerging models of corporate governance may reduce and prevent stress and burnout. Work is changing fundamentally, and this impacts workers’ (and managers’) health and well-being; that is why approaches in line with these changes are necessary. The COVID-19 pandemic has produced major changes in work organization. This may offer promising opportunities to reanalyze working conditions for a better control of occupational diseases and stress with all the benefits these improvements will bring for society and for individuals.

## 1. Introduction

Psychosocial risks (PSRs) have been studied for more than 50 years, and they are still uncontrolled at a global national or international level, as shown by relevant surveys [1,2,3]. Even if there are a few examples of success in reducing this risk in some companies [4], the global situation is still worsening, and the COVID-19 pandemic has even intensified this problem [5].

Solutions and strategies to prevent or reduce these risks have been proposed but appear to have been used in very limited specific situations and not accepted as a basic policy by decision-makers. Why? It is not possible to give a clear answer to this question, but a few hypotheses (among many others) can be proposed:

- There is a lack of awareness among decision-makers due to the fact that occupational safety and health (OSH) issues are not included in their priorities [1,6] and that OSH professionals have not found the appropriate way to inform and sensitize them;
- The solutions and strategies proposed up to now may be not convincing enough, being based on traditional approaches that are less and less efficient and not appropriate anymore in the new context of work [7].
- A lack of a common approach that includes with the OSH professionals, relevant partners such as economists, sociologists, managers, human resources directors, insurers, lawyers, politicians, etc. The experts remain usually in their own silo with more confidence in their peers than in other professionals [8].
- Economic difficulties leading to organizational adjustments (such as a reduction of the number of employees) to maintain the company may impact negatively on the working conditions [9].
- A lack of incentives to prevent PSRs whose detrimental effects (such as burnout, for instance) are very often not recognized as occupational diseases but only, at best, as work-related diseases (occupational phenomenon) not entering in the official and insurance statistics [10].
- No national or international assessment of the impact of PSRs through registers due to the difficulties to clearly define their nature and health effects [11].

The objectives of this paper are to present, in this broad context of societal and cultural changes, how the present shift in management paradigms may represent opportunities to reduce work-related diseases.

Although PSRs mainly concern mental health, they are related to other disease such as musculoskeletal disorders and lower-back pains [12].

In the first part of this article, relevant cultural and societal changes will be illustrated through three main issues: (1) evolution of the OSH field, (2) change in the nature of work, and (3) emerging models of governance. In its second part, a few examples of new management strategies that prevent and reduce PSRs will be presented and discussed.

Work is changing fundamentally and rapidly [13], and this impacts workers’ (and managers’) health and well-being. Moreover, the attitude towards work is also changing, especially for younger generations, and that is why approaches in line with these developments are necessary.

The COVID-19 pandemic has exacerbated PSRs and their health effects; therefore, this problem is a higher priority issue than ever before and is offering opportunities to introduce new avenues for better controlling occupational diseases with all the benefits these approaches will bring for the society and for the individuals.

## 2. Materials and Methods

The broad field of OSH and its evolution over the last 50 years has been followed carefully by the author and summarized in 2011 by stressing the unsuspected dimensions of this domain and their promising perspectives [14]. Based on this reference work some emerging concepts with emphasis on global health at work, quality of working life, core values that make work meaningful, and a few other ones will be presented along with related publications by international bodies.

The overview of the situation on the evolution of PSRs and theirs trends during the last decades is based on a non-exhaustive review of the scientific literature.

The emergence of new models of governance that may impact the prevention and reduction of PSRs has been reviewed through recent relevant basic studies and reference books.

Finally, a selection of examples of new management strategies has been carried out through different networks of companies adhering to governance policies in line with the evolution of our society such as environmental and societal responsibility, fair trade, sustainable development, quality of working life, etc.

## 3. The Society Today—Evolution and Changes

The present context that influences the way PSRs may be controlled is presented through three relevant issues illustrating significant societal and cultural changes.

### 3.1. Occupational Safety and Health

The International Commission of Occupational Health (ICOH) can be considered as the leading authority for promoting research and prevention as well as recognition of OSH professionals. Its code of ethics is internationally considered as a basic reference [15]. However, it is still focused almost exclusively on the detrimental effects of work and not on its positive effects such as well-being at work, quality of working life, or salutogenesis. Out of its 37 Scientific Committees, none are mainly dedicated to these beneficial aspects of work; they all look at potential negative impacts of work on workers’ health and not on the positive aspects of work and on well-being. This fact illustrates that the traditional objectives of OSH (prevention of accidents and diseases) remain predominant in this important community. However, other organizations, such as the WHO or the European Union [16], have launched in the early new century the concept of “Workplace Health Promotion” that stresses the importance of work organization, active participation of all actors, and the need for personal development, which is complementary to the OSH field and leads to a global approach of the work environment. This is illustrated by several international programs such as the “Healthy Workplace” model from the WHO [17], the Total Workers Health™ from the NIOSH in the USA [18], and the “Vision Zero” from the ISSA [19].

The detection and prevention of PSRs are parts of these international programs where some aspects of the changes in our society are considered. However, in the field, the effectiveness of the preventive measures has still not been clearly demonstrated since the epidemic is not yet controlled. Influence of relevant factors of influence such as new governance models, emerging visions of the companies, economic and legal challenges, cultural and societal changes, as well as expectations of younger generations have not really been investigated and assessed in relation to PSRs. This means that the OSH field has to enlarge its scope in research, education, and training, as well as in the development of preventive strategies integrated in the global context of the present changes.

### 3.2. Nature of Work

Work is changing rapidly and drastically and the traditional approaches to prevent health risks are becoming less and less appropriate. ILO has set up a Worldwide Commission for the future of work to ensure that programs remain human-centered [20] and the French National Research and Safety Institute has investigated the new methods of production and their impacts on OSH for the next decades [21].

Among the important changes that have to be considered, a few relevant ones will be shortly presented as illustrative examples that may be inter-related:

- Digitalization that includes robotization and artificial intelligence (AI) creates new working environments and new interactions such as the ones with collaborative robots that imply new ergonomic and psychologic approaches [22]. A lot of technological and virtual aspects of digitalization represent new challenges for detecting and preventing negative health effects, including PSRs.
- Holding different jobs at the same time (slashing) is becoming more and more usual, not only as a necessity to earn enough money to survive but also to satisfy wishes for variety and new experience for younger generation [23]. Since each job has its specific threats to health, the task of OSH professionals to prevent any harm is becoming more and more complex;
- Uberization allows, through a digital platform, to connect directly to providers of a service to customers without middle persons. This very convenient practice for the customers creates problems for the service providers who are often considered as self-employed workers without any social protection from the owner of the platform who takes a commission on each service and does not usually assume the costs of the social protection of the service providers [24]. Such a situation may initiate psychic stress among the concerned people.
- Teleworking (home working–remote working) has exploded during the COVID-19 pandemic and has had positive and negative impacts. Among the positive aspects, flexibility and tailored work organization is prevailing followed by the suppression of commuting that spares time and protect the environment. The negative aspects mainly concern the lack of direct social interactions, the difficulties in organizing professional and private life, and the risk of burnout (too much commitment) and depression. It appears that this major change will remain after the pandemic at a lower scale, but higher than before. This creates a blurred boundary between professional and private activities that may impact the work–life balance, leading to increased PSRs [25];
- Invisible jobs are those that are not well-considered, are underpaid, despised, class-biased, etc., such as road crews, garbage collectors, housekeepers, and many others. Unpaid jobs are also invisible, such as domestic tasks; voluntary work for associations, communities, non-governmental organizations; lending assistance to needy people; etc. These jobs must be better recognized as being just as important as the other ones and cannot be ignored in a global OSH approach [26].

Besides this list that could be extended to many other changes, there are cultural trends that influence the way OSH problems are perceived. The protection of the environment that exists for a long time and is integrated in the “Corporate Social Responsibility” is becoming a priority for some government and large companies with the risk that some “green jobs” are considered clean and without hazards [27]. The social networks spreading news of uncontrolled quality are both useful (speed and large audience) and dangerous (fake news) and should be considered with discernment by selecting reliable sources of information. Globalization is also speeding up as well as participation within companies and with external partners (clients, suppliers, etc.). These rapid changes and disruptions imply agility [28].

### 3.3. New Paradigms in the Governance of Companies

There is growing evidence that traditional management models, based only on the economic performance of the companies, are not appropriate to ensure healthy working conditions since they cause suffering and illnesses among employees that do not feel recognized and respected, or even feel exploited. This dehumanization of work contributes to developing the search for new models where the human being recovers its central place in companies and organizations [29]. The concept of “Freedom-form company” was introduced by Isaac Getz [30] in 2009 and showed that a “liberating leader” can build, in collaboration with their team, a specific mode of organization fitted to the company’s culture, where autonomy and shared values (confidence, integrity, etc.) play quite a relevant role to increase the performance and cohesion of the team.

In this context, two visionary thinkers (R. Barrett and F. Laloux) have investigated new management models based on organizations “that have a soul”. Their studies have triggered the development of a lot of management systems that look for increased performance both at economic and quality-of-working-life levels. Their description is out of the scope of this paper, but interesting surveys can be found in the literature, either in the human resources field or in the management or economic science [30,31,32,33].

Richard Barrett worked for the World Bank from 1979 to 1985 as a consultant and joined the bank in 1986 as the Assistant for the Vice-President for Environmentally Sustainable Development. He conceived a model wherein he set a parallel between the individuals’ consciousness levels and those of the companies (organizations). He defined seven levels that correspond to the natural evolution of any individual: (1) viability, (2) relationships, (3) performance, (4) evolution, (5) alignment, (6) collaboration, and (7) contribution [34]. These levels were inspired by Abraham Maslow’s Hierarchy of Needs and tested over more than two decades of real-world experience with thousands of organizations [35].

Frederic Laloux received an MBA/Master of Business Administration) from INSEAD (famous French School of Management) and a degree in coaching from Newfield Network; he was also a former Associate Partner with McKinsey & Company. After about 10 years, he realized that his job did not correspond to his values. He retired from the company and spent three years to study organizations that have adopted more powerful—and soulful—management practices. His book *Reinventing Organizations* [36] is considered by many to be the most influential management book of the last decade. It has inspired thousands of organizations throughout the world to take a radical leap and adopt a whole different set of management principles and practice. Looking at the evolution of organizations, he developed a model called *Teal* by reference of the different colors attributed to the previous models. *Orange*, for instance, is attributed to the traditional hierarchical model still largely used in companies.

**Figure 1:**
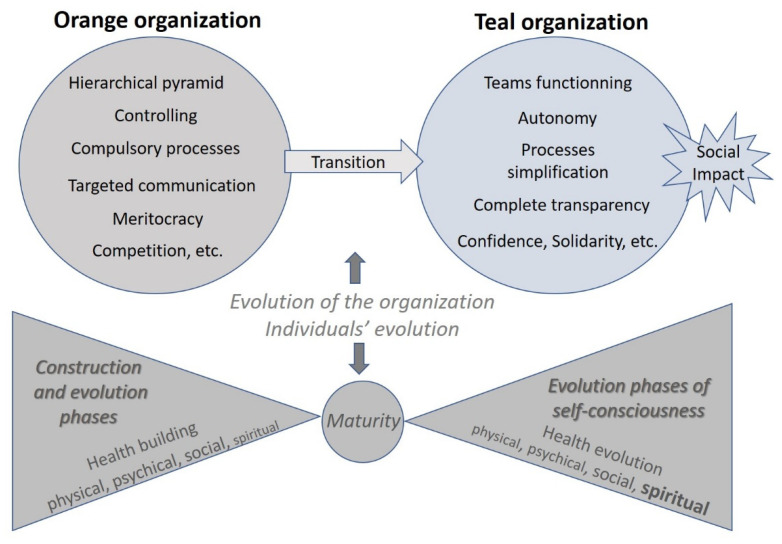
Figure inspired from Laloux and Barrett, illustrating the parallelism between the evolution of organizations and individuals.

There is a clear parallelism between Barrett’s and Laloux’ models, as illustrated in Figure 1.

The transition from the *Orange* to the *Teal* organization corresponds to the evolution of the organization according to the emerging values in our society and the individuals’ evolution corresponds to the phases of the construction of a human being who has first to build up his identity and his “global health” [37] until his maturity and then to develop his self-esteem and self-actualization (levels of consciousness) according to his values.

In the *Orange* organizations, the pyramidal hierarchy and performance indicators imply that as the organization is growing so do the number of levels of management to supervise, control, monitor, report the activities, productivity, output, etc. The system is becoming more and more complex, strict, and heavy due to the fact that any change has to be approved by the relevant superior. All these factors contribute to the decreased motivation, commitment, and initiative of all the staff members. The shift towards a *Teal* organization allows to fix these factors and opens new avenues for managing companies in a more efficient and human way, based on autonomy, transparency, confidence, and solidarity. A single example illustrates how these changes can impact on employees’ health: the coronary heart disease incidence is much higher for those who have low job control (autonomy) compared to those having a high job control [38].

From these basic concepts, a lot of different forms of organizations emerged of which the common objectives are increased economic and human performance based on shared values and adapted to the specific culture and mode of governance of each organization, where collaboration, autonomy, and mutual trust between employees and managers are fundamental. From an OSH perspective, such enterprises (small or large) have a better quality of working conditions and, therefore, less absenteeism, employee turn-over, and occupational health problems [39]. However, when the changes of governance are not based on these ethical principles but only on economic performance, negative and problematic issue emerges. Such companies argue that the new model of governance will also be beneficial for the employees, but they do not take care of their health and well-being and introduce pressure for more intensive and productive work, leading to burnout, depression, distrust, etc. [40]

In this context, the explosion of labels, certifications, standards, agreements, etc. illustrates what can be either a real motivation of companies, a search for an improved image or the following of a new fashion. The traditional ISO standards remain important references such as the new one on managing psychological health at work [41] but are more appropriate for the *Orange* models since they are still focused on the pyramidal hierarchy model.

## 4. Selected Examples of New Management Strategies Contributing to Reduce PSRs and to Increase Quality of Work

There is no uniform formula to prevent or reduce psychosocial risks. Measures have to be adapted to the size of the company, its culture, its vision and values, the type of leadership of the managers, and other specific factors related to the type of production or services, the location, the economic context, the OSH support, etc.

The number of companies implementing the new models of governance described above remain limited and not all are successful in their trials since the changes cause resistance and fear. In this chapter, only a few relevant examples will be presented, avoiding those who have apparently adopted a new governance strategy with the only main objective to increase productivity and improve the company image without true considerations for employees’ health and well-being.

### 4.1. A Pioneering Home Healthcare Organization

Buurtzorg, established in 2006 by Jos de Blok, is a national care company in the Netherlands providing domestic help to the sick and elderly, according to a nurse-led model of holistic care that revolutionized community care. Well described by Laloux [36], this company has gained international renown and has become a model thanks to its performance and its governance strategy. It employs 15,000 nurses and 950 teams that are autonomous and work much more efficiently than the traditional companies. It is one of the best examples of the shift from an *Orange* model to a *Teal* one. In the past, an imposed model led to the fact that the nurses’ tasks were specialized, standardized, timed, and controlled, which fits perfectly with the Orange system. However, the overall outcome has proved distressing both to patients and nurses. Patients lost their personal relationship with the nurses and the nurses suffered for working conditions they found degrading, especially due to the electronic registration device to control their activities. These practices were antagonist to their vocation to care for those in need. Even in the headquarter of such an organization, people did not find their work meaningful. 

The main characteristic of Buurtzorg is that the teams are self-managing and take on all the tasks that were previously fragmented in different departments and set up a network integrated to the local community with the doctors, pharmacies, and hospitals, and supported by coaches with no decision-making power. There is no leader, and important decisions are taken collectively; the most competent nurse for the decision to be taken is the accepted and recognized leader on that occasion. Vocation has been restored for the nurses. Patients and their families express their deep gratitude for the compassionately way they are treated.

The results obtained by Buutzorg are impressively excellent in terms of time to care for patients. In 2009, Ernst & Young found that 40% fewer hours are spent for patients compared to other traditional organizations and estimated that the savings for the Dutch social security system is close to EUR 2 billion. This apparent paradox between the fact that nurses spend more time with the patient and save 40% of the overall time dedicated to them is due to the fact that patients heal faster, gain more autonomy, and that one third of emergency hospital admission is avoided.

It is therefore not surprising that absenteeism, employee turnover, and work-related health problems are much lower in this company than in the other “traditional” ones [42].

This very short and summarized description of the “Buurtzorg model” [43] should not be interpreted as “the solution” for all the companies providing home care to the elderly and the sick. Challenges and difficulties to set up this model so that it runs smoothly should not be underestimated. It is a striking example of success in the Netherlands and in the 24 countries where it has been developed, but in countries where culture, social security systems, insurances, education and training, or traditions are different, such a model may not be applicable.

### 4.2. A Large International Group in the Field of Energy

Engie SA is a French multinational electric utility company, which operates in the fields of energy transition, electricity generation and distribution, natural gas, nuclear, renewable energy, and petroleum [44].

Founded in July 2008, this large leading firm was very successful during the first years but encountered difficulties and profitability losses as the pressure of the social responsibility about renewable energy and saving of energy increased. The top management decided to start fundamental changes and transformation of the company with a new vision: transition towards zero-carbon energy sources. This revolutionary evolution implied the mobilization of the collective intelligence, the development of new strategies, the employees’ training and education on new jobs and skills, as well as agility, so that everybody could feel concerned, responsible, and committed to cope with this huge challenge. Moreover, the emergence and flow of new ideas, and contact and collaboration with clients had to be improved; this was not possible with the traditional pyramidal model of management in place. Engie announced in 2019 the organization of its businesses around business lines and business units. The business unit “Global Energy Management” (5000 employees) explored the new concepts of management and work organization and inevitably arrived to holacracy (holacracy is a method of decentralized management and organizational governance, where authority and decision-making is distributed between self-organizing teams). The first feedbacks were very positive, and the company hired experts to internally develop an adequate model, which proved to be successful for innovation and employees’ well-being. The success of this business unit raised interest in the other units and illustrates the positive impact of these new forms of governance on the human resources and the performance of the company [45]. However, recently, the board of directors changed the leadership of the company, for political and economic reasons with unknown consequences.

### 4.3. A Brazilian Multi-Function Company

Semco Partners is a Brazilian company with different activities, making, among other things, industrial machinery such as mixers for pharmaceutical and candy companies, as well as cooling towers; it also provides consulting services for companies on different issues (environment, data centers, internet applications, human resources, etc.). Its owner, Ricardo Semler, has quite an innovative, nonconformist, and revolutionary concept of business based on participation and involvement by inspiring employees to assume ownership and responsibility and by giving up control [46]. The organization is based on some fundamental principles such as confidence, transparency, accountability, performance, honesty and fairness, participation, commitment, respect, etc. There are no organizational charts, no job titles, and no headquarters. Employees have the freedom to organize their work- and time schedule, determine their salary, select their manager, and express themselves openly. Barriers to unfair behavior are the transparency of the company’s financial information, peer pressure to keep budgets in line, the company’s culture and values, and inspiration from the CEO. Semler believes that challenging assumptions, rather than conforming to them, is the key to building an adaptive, creative organization. This model of organization, recognized as “industrial democracy”, is original and works probably thanks to Semler’s charisma.

The economic performance and profitability of Semco Partners are excellent. Customers are quite satisfied: 80% of the yearly revenues come from repeat customers. Employees’ satisfaction is demonstrated by a very low turnover (smaller than 1%) among 3000 employees. This means that these working conditions provide well-being and a good quality of working life for the company’s members.

Ricardo Semler and his company Semco gained international recognition for the groundbreaking alternative approach to management and organization they embodied. The Semco Style Institute (SSI), which was built upon this strong foundation, began operations in May 2016 in the Netherlands. Today, SSI is active in 11 countries and actively supports multiple organizations that range from fast-growing small-to-mid-sized enterprises to multinational corporate companies. SSI’s mission is to shape the future of work and they do so by helping organizations achieve more impact and better performance, with employees who are happier and more engaged [47].

### 4.4. An American Online Shoe and Clothing Retailer

The ZAPPOS Family of Companies, a subsidiary of Amazon.com, is a leader in online apparel and footwear sales by striving to provide shoppers the best possible service and selection. They carry millions of products from over 1000 footwear and apparel brands. Established in 1999, they are located in Las Vegas, NV, and have approximately 1500 employees, but continual growth makes that number ever-increasing. Zappos is using holacracy and adapts it to its needs. In this case, it is based on defined core values—whose clients’ satisfaction is an important one—and is built to focus on the work, rather than the people, but this does not mean that people are not important; on the contrary, each Zappos’ member finds his/her place, knows work he or she has to carry on, responsibility to assume and commitment to the company and its core values [48]. Leaders have a key role in promoting the company’s culture, innovation, and smooth work organization among employees, like a gardener that keeps the soil alive so that it becomes more fruitful.

The call center represents about 50% of the employees and the other ones are distributed in different “mission circles” that operate like micro-enterprises. There is still some hierarchy since there is a “general circle” that allocates budgets but does not interfere with the circle’s self-management. Tony Hsieh, who has been the CEO for about 20 years (up to 2020), was very concerned by the employees’ well-being and introduced new working methods and practices [49]. The company is very successful in terms of profitability and attraction since more than 30,000 job applications arrive every year.

### 4.5. A Canadian Company Specialized in Communication Technologies

Devicom is a small company (50 people) located in Chicoutimi, Canada, that provides small- and medium-sized businesses and large industries with the infrastructure necessary for unified and simplified communication, as well as adapted strategies anywhere, anytime, maintaining data security and systems reliability at moderate costs.

France Lavoie co-founded Devicom—a consulting group specializing in information technology—along with her spouse, Sylvian Duguay, in 1989. After 25 years, they realized that the company was no longer in a position to innovate, which had always been its mission. They diagnosed that the hierarchy had become too rigid and that it was time to reshape the organization: “That’s when I heard about holacracy, and I said to myself that was the answer to our slump,” wrote France [50]. This system of organizational governance is founded on the dissemination of decision-making mechanisms throughout the company; instead of resting on the shoulders of managers, responsibilities are shared by all employees, regardless of function. At Devicom, every project revolves around groups of individuals who work together and whose decisions have an immediate impact, both measurable and tangible, on the future of the company. “Now I hire based on a person’s understanding of and engagement with the company. That’s why we have a lot of longstanding self-employed workers and former entrepreneurs on our new team,” she explains. This drastic change in governance occurred in 2014 and made Devicom a pioneer in Canada, daring to shift from a traditional management style to a joint employee-management control promoting collective intelligence and harmony. Quality of working life and employees’ well-being is one of the key values of this company.

The company Devicom has been so successful that it was awarded “Prix Performance Québec 2018” by the Quebec government for its quality of management and its global achievement in 2018 at the Trade Show of Best Practices.

## 5. Discussion

The examples described above illustrate that the new governance models emerging in the last decades are promising for the reduction of PSRs and other risks since they give much more autonomy and empowerment to the employees and workers who are the first concerned with the occupational risks. These good examples should not hide the bad ones, where drift towards abuse and unethical behavior leads to manipulating workers or deviating from the original model [51].

Occupational health and safety science has recommended for a long time in one of its three main focuses that it should aim at “the development of work organizations and working cultures in a direction which supports health and safety at work and in doing so also promote positive social climate and smooth operation and may enhance productivity of the undertakings” [15]. However, research on the type of governance models and the impact they may have on the working conditions and workers’ health and well-being does not seem to have been much investigated, scientifically. This topic should become a multidisciplinary area of research where managers, human resources experts, sociologists, economists, lawyers, and occupational safety and health professionals can bring quite relevant contributions by working together. This type of approach, breaking the silos of the individual disciplines, is quite unusual due to the difficulties to enlarge the scope of its own discipline and due to the preference to remain between peers. This is true even inside a main discipline: in medicine, specialists in different areas have difficulties cooperating [52]. Therefore, this represents a challenge for the research aiming at better understanding why and how the governance models influence the prevention and control of the PSRs.

There is an agreement between all the studies on this topic that there is no single formula to prevent or reduce psychosocial risks [53]. If it clearly appears that the management models, styles, and strategies play quite a relevant role, no rules or directives can be given to implement specific measures. Each company has to find its own way to build up its vision and its organization that promote innovation, economic performance, social responsibility, and employees’ and workers’ satisfaction by taking into account its values, culture, and quality of its production or services.

## 6. Conclusions

This paper does not claim to be a thorough investigation on the ways new governance models impact positively on the prevention of PSRs and other occupational risks, but it brings relevant elements that show these new management strategies offer real opportunities to improve working conditions and workers’ health and wellbeing.

This paradigm change in the governance represents a challenge for CEOs and managers and as any innovation may be unsuccessful, leading to disappointment for the whole staff as well as stress and depression. Examples of such failures are numerous. Frequent cases are linked to the CEO’s charisma that is successful as long as he manages the society, but when he leaves, the usual human behavior reverts to competition, selfishness, inflated egos, etc., which destroy harmony and success [54].

The COVID-19 pandemic that has destabilized the usual way of working may be an opportunity to cope with these challenges without delay [55].

To conclude, it is clear that this essential field of human activities deserves more research and attention since it will open new avenues for prevention and health promotion, not only in occupational health but also in public health.

## Data Availability

Not applicable.

## References

[1] European Agency for Safety and Health at Work Management of Psychosocial Risks in European Workplaces: Evidence from the Second European Survey of Enterprises on New and Emerging Risks (ESENER-2)-European Risk Observatory Report. https://osha.europa.eu/en/publications/second-european-survey-enterprises-new-and-emerging-risks-esener-2-overview-report.

[2] Lesuffleur T., Chastang J.F., Cavet M., Niedhammer I. (2015). Psychosocial Work Factors and Self-Reported Health in the French National SUMER Survey. Sante Publique.

[3] Proper K.I., van Oostrom S.H. (2019). The effectiveness of workplace health promotion interventions on physical and mental health outcomes: A systematic review of reviews. Scand. J. Work Environ. Health.

[4] Rosario S., Fonseca J.A., Nienhaus A., Torres da Costa J. (2016). Standardized assessment of psychosocial factors and their influence on medically confirmed health outcomes in workers: A systematic review. J. Occup. Med. Toxicol..

[5] Giorgi G., Lecca L.I., Alessio F., Finstad G.L., Bondanini G., Lulli L.G., Arcangeli G., Mucci N. (2020). Covid-19 Related Mental Health Effects in the Workplace: A Narrative Review. Int. J. Environ. Res. Public Health.

[6] Sivaram P. (2019). Lack of General Safety Awareness in the Society. AIRSC Academy of Safety and Disaster Management. https://www.researchgate.net/publication/330117124_Lack_of_General_Safety_Awareness_in_the_Society.

[7] Magnavita N., Chirico F. (2020). New and Emerging Risk Factors in Occupational Health. Appl. Sci..

[8] Senge P.M. (2004). The Fifth Discipline: The Art and Practice of the Learning Organization.

[9] Frone M.F., Blais A.R. (2020). Organizational Downsizing, Work Conditions, and Employee Outcome: Identifying Targets Workplace Intervention among Survivors. Int. J. Environ. Res. Public Health.

[10] WHO News (2019). Burn-Out An “Occupational Phenomenon”: International Classification of Diseases. https://www.who.int/news/item/28-05-2019-burn-out-an-occupational-phenomenon-international-classification-of-diseases.

[11] Leka S., Jain A. (2010). Health Impact of Psychosocial Hazards at Work: An Overview.

[12] Szerla M., Ortenburger D., Kluszczyński M., Wyszomierska J. (2017). Exercise and psychological factors in low back pain. Phys. Activ. Rev..

[13] ILO (2019). Occupational Safety and Health. Safety and Health at the Heart of the Future of Work Building on 100 Years of Experience.

[14] Guillemin M. (2011). Les Dimensions Insoupçonnées de la Santé au Travail.

[15] International Commission on Occupational Health (ICOH) (2014). ICOH International Code of Ethics for Occupational Health Professionals.

[16] European Network of Workplace Health Promotion (2018). Luxembourg Declaration on Workplace Health Promotion in the European Union.

[17] Burton J. (2010). WHO Healthy Workplace. Framework and Model: Background and Supporting Literature and Practice.

[18] Hudson H.L., Nigam J.A.S., Sauter S.L., Chosewood L.C., Schill A.L., Howard J. (2019). Total Worker Health™.

[19] International Social Security Association (2017). Vision ZERO, 7 Golden Rules for Zero Accidents and Healthy Work; A Guide for Employers and Managers.

[20] ILO (2019). Global Commission on the Future of Work. Work for a Brighter Future.

[21] Institut National de Recherche et de la Sécurité (INRS) (2016). Modes et Méthodes de Production en France en 2040: Quelles Conséquences Pour la Santé et la Sécurité au Travail?.

[22] European Agency for Safety and Health at Work, (OSHA-Europe) (2021). Impact of Artificial Intelligence on Occupational Safety and Health.

[23] Hong Kong Baptist University, (HKBU) (2018). Slash Generation is Coming. HKBU News.

[24] Towers-Clark C. (2019). The Uberization of Work: Pros and Cons of the Gig Economy.

[25] Work Mind, Content Team (2021). Blurred Boundaries between Work and Home Life: New Data Suggest Emotional and Physical Drift.

[26] Quinlan M. (2015). The Effects of Non-Standard Forms of Employment on Worker Health and Safety.

[27] European Agency for Safety and Health at Work (OSHA-Europe) (2021). Workers’ Safety and Health in Green Jobs.

[28] Jesuthasan R., Boudreau J. (2021). Work without Jobs: How to Reboot Your Organization’s Work Operating System.

[29] Christoff K. (2014). Dehumanization in organizational settings: Some scientific and ethical considerations. Front. Hum. Neurosci..

[30] Carney B.M., Getz I. (2016). Freedom, Inc.: How Corporate Liberation Unleashes Employee Potential and Business Performance.

[31] Matthey-Doret D., Pétermann M. (2018). Nouveau Paradigme Organisationnel et Managérial: État des Lieux en Suisse.

[32] Bretones L., Pinault P., Trannoy O. (2020). L’Entreprise Nouvelle Génération.

[33] Destatte P. (2019). Some “New” Governance Models for Europe and the United States. Cadmus.

[34] Barrett R. (1998). Liberating the Corporate Soul: Building a Visionary Organization.

[35] Wikipedia. Maslow’s Hierarchy of Needs. https://en.wikipedia.org/wiki/Maslow%27s_hierarchy_of_needs.

[36] Laloux F. (2014). Reinventing Organizations: A Guide to Creating Organizations Inspired by the Next Stage of Human Consciousness.

[37] Guillemin M. (2019). The New Dimensions of Occupational Health. Health.

[38] Marmot M.G., Bosma H., Hemingway H., Brunner E., Stansfeld S. (1997). Contribution of job control and other risk factors to social variations in coronary heart disease incidence. Lancet.

[39] Tamers S.L., Streit J.M.K., Swanson N., Hammer L. (2021). The Role of Organizational Design in the Future of Work. NIOSH Science Blog. http://blogs.cdc.gov/niosh-science-blog/2021/01/12/future-of-work-design/.

[40] Bernstein E., Bunch J., Canner N., Lee M. (2016). Beyond the Holacracy Hype. Harv. Bus. Rev. (HBR).

[41] ISO Technical Committee (2021). ISO/TC 283 Occupational Health and Safety. ISO 45003:2021 Occupational Health and Safety Management, Psychological Health and Safety at Work: Guidelines for Managing Psychosocial Risks.

[42] Vieira H. (2016). The Paradox of Bullied and Frightened Workers Delivering Quality Care; London School of Economics and Political Science Blog. https://blogs.lse.ac.uk/businessreview/2016/11/28/the-paradox-of-bullied-and-frightened-workers-delivering-quality-care/.

[43] Buurtzorg International The Buurtzorg Model. https://www.buurtzorg.com/about-us/buurtzorgmodel/.

[44] ENGIE Act with ENGIE: What If the Common Good Was Everyone’s Business?. https://www.engie.com/en/act-with-engie.

[45] Kochner I., Bretones L., Pinault P., Trannoy O. (2020). Regards de pionniers, 1.2: Isabelle Kocher, ex-Direcrice générale du groupeENGIE. L’Entreprise Nouvelle Génération.

[46] Epic Work Epic Life Semco, Insanity that Works: A Philosophy of Participation and Involvement. https://epicworkepiclife.com/semco-insanity-that-works/.

[47] Semco Style Institute Shaping the Future of Work. https://semcostyle.com/.

[48] Zappos Insight Culture Drives Success. https://www.zapposinsights.com/.

[49] Hsieh T. (2010). Delivering Happiness: A Path to Profits, Passion and Purpose. Zappos.com. https://allbooksworld.com/delivering-happiness-by-tony-hsieh-epub-download/.

[50] (2017). Devenir Entrepreneur. Devicom: Evicom a Local Innovator, France Lavoie’s Story. Entrepreneur Stories. https://devenirentrepreneur.com/en/articles/entrepreneur-stories/devicom-local-innovator.

[51] Marnet O. (2008). Behaviour and Rationality in Corporate Governance. Int. J. Behav. Account Financ..

[52] Stratil J.M., Rieger M.A., Völter-Mahlknecht S. (2017). Cooperation between general practitioners, occupational health physicians and rehabilitation physicians in Germany: What are problems and barriers to cooperation? A qualitative study. Int. Arch. Occup. Environ. Health.

[53] ANACT (Agence Nationale pour l’Amélioration des Conditions de Travail) (2015). Synthèse documentaire sur l’entreprise libérée. Étude et Rapport. L’entreprise Libérée.

[54] Roussange G. (2020). Vers un Plan de Rupture Conventionnelle Collective à la Fonderie FAVI. ECO121, Blog. https://www.eco121.fr/fonderie-favi-hallencourt-somme/.

[55] Baez Camargo C. Rethinking Governance in the Times of the Covid-19 Pandemic. Basel Institute on Governance, Blog. 2020. https://baselgovernance.org/blog/rethinking-governance-times-covid-19-pandemic.

